# Regenerative endodontics: current concepts, clinical challenges, and emerging therapies

**DOI:** 10.1590/1807-3107bor-2026.vol40suppl1063

**Published:** 2026-08-21

**Authors:** Patricia de Andrade RISSO, Ana Paula PORTES-ZENO, Alexandre Guimarães dos SANTOS, Alice Corrêa SILVA-SOUSA, Rodrigo ARRUDA-VASCONCELOS, Emmanuel João Nogueira Leal SILVA, Carla Renata SIPERT

**Affiliations:** (a) Universidade Federal do Rio de Janeiro - UFRJ, School of Dentistry, Department of Dental Clinics (Endodontics), Rio de Janeiro, RJ, Brazil; (b) Universidade Federal do Rio de Janeiro - UFRJ, School of Dentistry, Department of Pediatric Dentistry and Orthodontics, Rio de Janeiro, RJ, Brazil; (c) Universidade de São Paulo - USP, School of Dentistry, Department of Dentistry, São Paulo, SP, Brazil; (d) Universidade de São Paulo - USP, School of Dentistry of Ribeirão Preto, Department of Restorative Dentistry, Ribeirão Preto, SP, Brazil; (e) Universidade Estadual do Rio de Janeiro - UERJ - Deparment of Endodontics, Rio de Janeiro, RJ, Brazil

**Keywords:** Regenerative Endodontics, Dental Pulp Necrosis, Root Canal Therapy

## Abstract

Regenerative endodontics has transformed the management of immature necrotic teeth by offering the potential to improve root structure rather than merely induce apical closure. Over the past two decades, considerable progress has been made in case selection, disinfection protocols, irrigation strategies, intracanal medicaments, scaffold design, and biologically based tissue engineering approaches. Despite these advances, key challenges remain, including protocol standardization, variability in clinical outcomes, limited high-quality clinical evidence, and the gap between promising laboratory findings and routine clinical implementation. This narrative review summarizes the recent literature on regenerative endodontics, focusing on contemporary clinical protocols and their limitations, prognostic factors, treatment outcomes, and emerging therapeutic strategies. Current evidence supports regenerative procedures that involve careful case selection, appropriate scaffold formation, well-sealed coronal restorations, and effective disinfection that preserves stem cell viability. While these protocols promote infection control, periapical healing, and tooth survival, continued root maturation remains variable, and existing therapies primarily result in tissue repair rather than true regeneration of a functional pulp-dentin complex. Additionally, there are several limitations to the widespread clinical adoption of advanced regenerative therapies, including outcome measures, alongside regulatory, manufacturing, and logistical challenges. Bridging the gap between innovation and clinical practice will require continued advances in tissue engineering, as well as more robust clinical evidence and standardized treatment protocols.

## Introduction

Endodontic treatment of necrotic teeth with open apices has traditionally relied on apexification procedures, which provide apical closure either through long-term calcium hydroxide therapy^1^ or by placing an artificial apical barrier with calcium silicate-based materials.^2^ Contemporary calcium silicate-based cements (particularly mineral trioxide aggregate [MTA] and related bioceramics) have largely replaced long-term calcium hydroxide because they shorten treatment time, reduce clinical visits, and achieve comparable clinical outcomes^3^. However, irrespective of the specific apexification technique employed, these procedures fail to promote continued root development or dentinal wall thickening, leaving immature teeth more susceptible to fracture.^1^,^3^,^4^

Regenerative endodontic procedures (REPs) for immature necrotic teeth have gained prominence for their potential to support continued root development rather than only inducing apical closure.^5^ Unlike apexification, REPs aim to establish a biological environment in the root canal space through interactions among endogenous stem cells, growth factors, bioactive molecules, and scaffold materials.^6^,^7^

While REPs offer the possibility to improve root structure, apexification remains a predictable, well-established treatment option for immature necrotic teeth.^8^ Calcium silicate-based apical barrier techniques shorten treatment times and provide predictable clinical outcomes; in contrast, REPs uniquely offer the potential for further root development, although the extent of this response is inconsistent.^9^ However, evidence demonstrating that REPs produce superior long-term clinical outcomes compared with contemporary apexification techniques remains limited.^10^ Therefore, treatment selection should consider not only root maturation potential but also patient- and tooth-related factors, as well as the strength of current clinical evidence supporting each therapeutic approach.^2^

This balance between biological potential and clinical predictability highlights central challenge of regenerative endodontics.^11^ Despite substantial advances over the past two decades, current REPs remain only partially predictable. Clinical success is generally high for clinical symptoms and periapical healing;^12^,^13^,^14^,^15^ however, continued root maturation,^2^ tissue composition,^16^ and true pulp-dentin regeneration^16^,^17^ are highly variable.^10^,^18^ In addition, the majority of emerging regenerative strategies have demonstrated promising results in laboratory and preclinical studies, with limited translation into routine clinical practice.^19^,^20^ Accordingly, this review outlines the current state of regenerative endodontics, critically discusses the major clinical challenges that constrain treatment predictability, and examines emerging therapeutic strategies that may improve outcome predictability.

## Methods

This narrative review provides an updated summary of REPs, focusing on clinical protocols, prognostic factors, treatment outcomes, current limitations, and emerging therapeutic strategies. A literature search was conducted in the PubMed database using the MeSH terms "Regenerative Endodontics", "Hydrogels", and "Stem Cells", together with the keywords "pulp revascularization", "immature necrotic tooth", "scaffold", and "growth factor", combined with Boolean operators. Original studies, systematic reviews, meta-analyses, clinical guidelines, consensus statements, and landmark original studies were selected based on their relevance to the review topics, with a particular emphasis on recent high-level evidence and clinically applicable findings when available.

### Clinical protocols for REPs in immature necrotic teeth

Despite differences in irrigating solutions, intracanal medicaments, and scaffold materials, contemporary regenerative endodontic protocols consistently achieve high rates of resolution of clinical signs and symptoms, periapical healing, and tooth survival.^13^,^14^,^15^ However, outcomes related to continued root maturation remain inconsistent, reflecting the limited predictability of current regenerative procedures.^2^,^12^

Although treatment protocols continue to evolve, current clinical recommendations are primarily based on guidelines issued by the American Association of Endodontists (AAE)^21^ and the European Society of Endodontology (ESE)^22^ (Table 1), alongside evidence from systematic reviews^9^,^12^,^13^,^18^ and clinical studies.^5^,^14^,^15^ Both the AAE^21^ and the ESE^22^ recommend a biologically based cell-homing approach that prioritizes effective canal disinfection while preserving stem cell viability, followed by induction of apical bleeding to create an autologous scaffold that recruits endogenous stem cells.

**Table 1:** Consensus and key differences between the American Association of Endodontists (AAE) and the European Society of Endodontology (ESE) recommendations for regenerative endodontic procedures.

Clinical step	Consensus (AAE and ESE)	Key differences	Clinical implication
Case selection	REPs are recommended for immature permanent teeth with pulp necrosis after appropriate case selection. Teeth should be restorable and suitable for long-term follow-up.	ESE provides more detailed recommendations regarding informed consent, patient expectations, and restorability.	Careful case selection remains fundamental, but current evidence suggests that many traditional prognostic factors should not be considered absolute contraindications.
Mechanical instrumentation	Minimal or no mechanical instrumentation is recommended to preserve dentinal walls and the apical stem-cell niche.	AAE allows gentle instrumentation when clinically indicated, whereas ESE places greater emphasis on avoiding instrumentation whenever possible.	The extent of instrumentation should be individualized according to root development and clinical presentation, as robust evidence supporting one approach over the other remains limited.
Irrigation	Low-concentration sodium hypochlorite followed by 17% EDTA is recommended to balance disinfection and stem-cell preservation.	Minor differences exist regarding irrigation sequence and procedural details.	This combination currently represents the best-supported irrigation protocol for contemporary REPs.
Intracanal medication	Intracanal medication is recommended between appointments to improve canal disinfection.	AAE recommends calcium hydroxide or low-concentration TAP/DAP, whereas ESE preferentially recommends calcium hydroxide.	Calcium hydroxide and antibiotic pastes remain the principal options, each presenting specific biological and clinical trade-offs.
Second appointment	Regenerative procedures should only proceed after resolution of clinical signs and symptoms of infection.	ESE provides more explicit recommendations regarding management of persistent inflammation before continuing treatment.	Effective infection control remains the primary prerequisite for regenerative procedures.
Scaffold	Induction of a blood clot is considered the standard scaffold for contemporary REPs.	AAE explicitly recognizes PRP and PRF as alternative scaffolds; ESE focuses primarily on blood clot induction.	Current evidence does not consistently demonstrate superior clinical outcomes for platelet concentrates compared with blood clot.
Coronal barrier	Calcium silicate-based cements are recommended to seal the scaffold before definitive restoration.	ESE specifically discusses newer tricalcium silicate materials (e.g., Biodentine® and other bioceramics) in greater detail.	Long-term treatment success appears to depend more on an effective coronal seal than on the specific calcium silicate-based material selected.
Definitive restoration	Immediate placement of a durable coronal restoration is recommended following scaffold sealing.	No clinically relevant differences.	Prevention of coronal microleakage is essential for maintaining the regenerative microenvironment.
Follow-up and outcome assessment	Long-term clinical and radiographic follow-up is recommended.	ESE proposes a more comprehensive evaluation, including patient acceptance, discoloration, periodontal ligament formation, and esthetics, whereas AAE focuses primarily on healing, root maturation, and pulp sensibility.	Clinical success should be interpreted using multiple outcome measures, recognizing that healing, root maturation, and pulp sensibility do not necessarily indicate true pulp-dentin regeneration.

**Figure 1:**
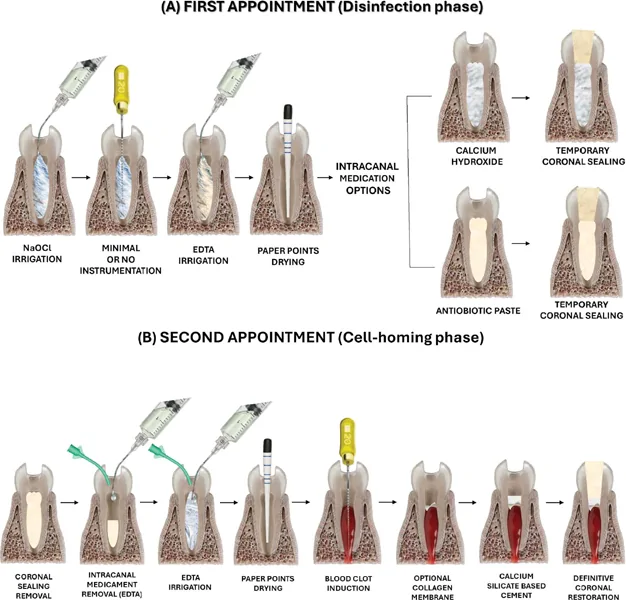
Contemporary clinical protocol for regenerative endodontic procedures.

Regenerative endodontic treatment is generally performed over two appointments. The first visit focuses on infection control with minimal or no mechanical instrumentation. This involves irrigating the canal with biologically compatible concentrations of sodium hypochlorite followed by ethylenediaminetetraacetic acid (EDTA), before placing an intracanal medicament^21^,^22^ (Figure 1A). During the second appointment, bleeding is induced into the disinfected canal, forming a blood clot that serves as an autologous scaffold for endogenous stem cell recruitment. This scaffold is then covered with a bioceramic material and restored with an effective coronal seal to minimize reinfection^21^,^22^ (Figure 1B). The following sections examine the evidence supporting each stage of contemporary REPs protocols, including disinfection, intracanal medication, scaffold selection, and emerging therapeutic approaches.^23^,^24^,^25^

### Case selection and prognostic factors

REPs are indicated for permanent teeth that exhibit pulp necrosis and immature apices, regardless of the presence of periapical lesions, provided that the tooth can be permanently restored without an intraradicular post and that no systemic contraindications exist.^21^ Treatment planning should also consider the ability of the patient or their caregiver to comply with the treatment and follow-up protocol.^21^

Several preoperative factors have been proposed as potential predictors of REPs success, including patient age, root morphology, root development stage, apical foramen diameter, the presence of periapical lesions, and the etiology of pulp necrosis.^2^,^11^ Favorable outcomes have been reported for a wide range of apical sizes, including in mature teeth; the minimum apical diameter required for success therefore remains controversial, as favorable outcomes have been reported across a wide range of apical sizes, including in mature teeth.^26^,^27^ However, recent systematic reviews have highlighted the uncertain clinical impact of these factors, reporting that the literature does not consistently link variables such as the etiology of pulp necrosis,^28^,^29^,^30^ apical foramen diameter,^31^,^32^ patient age, root development stage, and the presence of periapical lesions with either the clinical or radiographic outcomes of REPs.^33^ While very low-certainty evidence suggests that trauma-induced pulp necrosis, tooth group (incisors), and apical lesions may be associated with less favorable root development, these findings have not been consistently reproduced.^33^

From a clinical perspective, these findings indicate that preoperative factors should not be interpreted as independent predictors of treatment success or used in isolation to determine case selection. Instead, they should be integrated into a comprehensive clinical assessment that includes the tooth's restorability, the feasibility of achieving adequate disinfection, patient compliance, and the clinician's experience. Clarification about the prognostic value of preoperative factors along with other potential variables will require well-designed prospective studies with standardized protocols and appropriate case stratification.^33^

(A) First appointment (disinfection phase). Schematic illustration of the first clinical appointment, highlighting conservative canal disinfection utilizing minimal or no instrumentation, irrigation with sodium hypochlorite and EDTA, placement of intracanal medication, and temporary coronal sealing. (B) Second appointment (cell-homing phase). Schematic illustration of the second clinical appointment, including removal of the intracanal medicament, EDTA irrigation, induction and stabilization of the blood clot as an autologous scaffold, optional collagen membrane placement, sealing with a calcium silicate-based cement, and definitive coronal restoration (adapted from the AAE position statement).

### Disinfection (minimal instrumentation, irrigating solutions and methods, and intracanal medications)

Successful regenerative endodontic procedures depend on striking a careful balance between effective canal disinfection and preservation of the biological conditions essential for regeneration.^34^ Residual infection and persistent inflammation are among the primary biological factors that undermine the predictability of REPs.^5^,^13^,^35^ Resistant biofilms and their antigens modulate the release of inflammatory mediators and compromise the viability, proliferation, and differentiation of stem cells from the apical papilla (SCAP), adversely affecting their regenerative potential.^36^,^37^

Experimental data also indicate that persistent bacterial contamination favors differentiation toward an osteogenic rather than an odontogenic phenotype, leading to the formation of bone- or cementum-like tissues instead of a functional pulp-dentin complex.^16^ This biological response appears to be at least partly mediated by inflammatory mediators such as Tumor Necrosis Factor (TNF)-α, Interleukin (IL)-1β, IL-6, and angiotensin II, all of which inhibit SCAP differentiation and mineralization and the expression of odonto/osteogenic genes.^38^,^39^

Consequently, rather than simply maximizing antimicrobial activity, contemporary REPs protocols aim to achieve sufficient microbial reduction while preserving both stem cell viability and the regenerative potential of the apical tissues.^34^ This biological balance informs the irrigation protocols, intracanal medicaments, and adjunctive disinfection strategies detailed in the following sections.

### Minimal or no instrumentation

Mechanical instrumentation aids root canal disinfection by disrupting bacterial bio films and facilitating the penetration and exchange of irrigating solutions along the canal walls.^40^,^41^ Unlike conventional root canal treatment, REPs seek to preserve the structural integrity of immature teeth and the viability of stem cells in the apical tissues.^10^ Excessive dentin removal should therefore be avoided, particularly in teeth with thin, fragile root walls.^10^

For this reason, some regenerative protocols generally advocate minimal^7^,^14^ or no^21^,^22^,^42^ mechanical instrumentation and rely primarily on chemical disinfection to reduce microbial burden while avoiding additional weakening of the root structure.^43^,^44^ Evidence regarding the role of mechanical instrumentation in REPs is limited. Published clinical protocols show considerable variability in root canal preparation, ranging from no instrumentation to minimal mechanical instrumentation.^44^ When instrumentation is deemed necessary, the decision should be individualized based on the stage of root development, the root canal anatomy, and the anticipated benefits of biofilm disruption.^7^ In such cases, instrumentation should be limited to the cervical and middle thirds of the canal, using flexible nickel-titanium instruments or passive agitation systems to minimize unnecessary dentin removal.^45^.

### Irrigating solutions and methods

Disinfection protocols in REPs must simultaneously maximize microbial reduction, preserve apical stem cells' viability and functionality, and stimulate the release of dentin-derived growth factors^16^,^45^. While potent antimicrobial agents are required for effective disinfection, successful regeneration depends on maintaining a microenvironment that supports stem cell survival, migration, and differentiation.^45^,^46^ Consequently, irrigation protocols for REPs should balance antimicrobial efficacy with biological compatibility.

Irrigation with 1.5-3% sodium hypochlorite (NaOCl), followed by a final rinse with 17% EDTA, is the recommended irrigation protocol for REPs based on the current international statements.^21^,^22^ NaOCl is the irrigant of choice because of its broad antimicrobial activity and tissue-dissolving capacity, although higher concentrations (e.g., 6%) should be avoided because they exert detrimental effects on stem cell viability.^45^,^47^ EDTA removes the inorganic component of the smear layer, partially reverses NaOCl-induced cytotoxic effects, and promotes the release of bioactive molecules (e.g., transforming growth factor-β1 [TGF-β1]) from the dentin matrix.^17^,^47-49^ Although bacterial contamination can lessen the biological effects of EDTA-mediated growth factor release,^49^ at present, the combination of NaOCl (1.5-3%) and 17% EDTA provides the most favorable balance among antimicrobial activity, stem cell preservation, and biological signaling.^22^,^34^,^47^

The use of 2% chlorhexidine as an irrigant has also been proposed,^50^ although its application in REPs remains controversial. While chlorhexidine possesses antimicrobial activity, in vitro studies have demonstrated that it exerts significant cytotoxic effects on stem cells,^51^,^52^ along with prolonged residual effects due to its substantivity.^52^ Furthermore, because chlorhexidine lacks tissue-dissolving capacity, its use as the primary irrigant in REPs is not currently recommended.^34^ Alternative chelating agents such as citric acid have also been investigated, demonstrating smear layer removal comparable to that achieved using EDTA, although its ability to induce growth factor release appears less consistent.^53^

With the aim of further optimizing the balance between antimicrobial activity and biological compatibility, several other irrigating solutions have recently been investigated. Calcium hypochlorite [Ca(OCl)2] has demonstrated antimicrobial efficacy comparable to NaOCl while exhibiting greater biocompatibility in laboratory studies. Coaguila-Llerena et al.^54^ reported that, compared with 1.5% NaOCl, 1.5% Ca(OCl)2 caused less structural damage to the apical papilla and promoted greater stem cell viability, proliferation, and osteogenic differentiation. When evaluated as an intracanal medicament, calcium hypochlorite has also exhibited superior antimicrobial activity against *Enterococcus faecalis* compared with triple antibiotic paste and calcium hydroxide.^55^ However, these findings are currently limited to laboratory investigations, and clinical evidence supporting the routine use of calcium hypochlorite in REPs remains unavailable.

Biologically active chitosan-based irrigants have also attracted growing interest because of their antimicrobial properties and favorable biocompatibility. Chitosan nanoparticles and 0.2% chitosan solutions demonstrate lower cytotoxicity than conventional irrigants while enhancing cellular responsiveness to TGF-β1.^56^ Additionally, combining chitosan nanoparticles with EDTA and irrigation activation techniques appears to increase growth factor release compared with EDTA alone.^57^ Although these findings are encouraging, they are currently only supported by in vitro evidence and therefore cannot yet justify clinical recommendations.

Adjunctive irrigation activation techniques have also been proposed to improve disinfection while maintaining the use of low NaOCl concentrations,^58^ including passive ultrasonic irrigation (PUI); negative-pressure irrigation systems (e.g., EndoVac); sonic activation devices (e.g., EndoActivator, Vibringe, and EDDY); laser-assisted irrigation techniques, such as photon-induced photoacoustic streaming (PIPS) and shock wave-enhanced emission photoacoustic streaming (SWEEPS); photodynamic therapy (PDT); and supplementary instruments such as the XP-Endo Finisher and the Self-Adjusting File (SAF).

Of these approaches, PUI is supported by the greatest body of experimental evidence and is already widely incorporated into conventional endodontic practice. Laboratory studies indicate that PUI enhances irrigant penetration,^59^ improves root canal disinfection^60^ and intracanal medicament removal,^61^ and increases EDTA-mediated growth factor release without additional dentin removal.^62^,^63^,^64^ Under experimental conditions, SAF instrumentation systems have also demonstrated improved bacterial reduction during chemomechanical preparation.^65^ However, direct clinical evidence supporting the use of irrigation activation systems in REPs remains limited.^66^

Laser-assisted activation methods, such as SWEEPS, have also demonstrated promising laboratory results, including in the form of enhanced irrigant dynamics,^67^ the removal of accumulated hard-tissue debris,^68^ microbial biofilms,^69^ intracanal medicament,^61^ and the enhanced adhesion and viability of dental pulp stem cells.^70^ Despite these promising findings, the results of experimental studies have been inconsistent,^71^ and systematic reviews of clinical studies evaluating irrigation activation methods have reported substantial heterogeneity, precluding robust clinical recommendations.^72^ Therefore, the incorporation of these techniques into routine regenerative endodontic procedures cannot currently be recommended. Moreover, experimental studies have identified apical irrigant extrusion as a potential concern associated with some laser-assisted activation methods in immature teeth with open apices.^73^

Overall, while several novel irrigants and activation technologies exhibit promising biological^54^,^56^,^57^,^62-64^,^70^ and antimicrobial properties,^55^,^60^,^65^,^69^ current clinical recommendations continue to rely primarily on irrigation with 1.5-3% NaOCl followed by 17% EDTA.^21^,^22^ Future well-designed clinical trials are required before these emerging strategies can be incorporated into evidence-based regenerative endodontic protocols.

### Intracanal medicaments

Intracanal medicaments are used as an adjunct to disinfection, typically for 1-4 weeks or until clinical signs and symptoms of infection resolve.^21^,^22^ Their primary objective is to further reduce the microbial load while creating conditions that favor stem cell survival and subsequent tissue repair.^41^,^58^

The ideal intracanal medicament should therefore offer effective antimicrobial activity without compromising stem cell viability or the release of dentin-derived bioactive molecules.^51^,^58^ Achieving this balance remains a major biological challenge to the implementation of REPs.^41^,^58^

The medicaments currently recommended for REPs include triple antibiotic paste (TAP), double antibiotic paste (DAP), and calcium hydroxide [Ca(OH)2]-based pastes.^21^,^22^ Triple antibiotic paste (ciprofloxacin, metronidazole, and minocycline) exhibits excellent antimicrobial activity and has been widely used in regenerative protocols.^5^,^14^ However, its biological effects are concentration-dependent, and high concentrations may reduce stem cell viability, impair the release of growth factors from dentin, and compromise tissue regeneration.^58^ Additionally, minocycline is strongly associated with tooth discoloration. Low-concentration TAP (1-5 mg/L) has been recommended for this reason; when used clinically, the medicament should be placed below the cementoenamel junction or after sealing the coronal dentin to minimize discoloration.^21^

DAP was developed primarily to eliminate minocycline-related discoloration. Despite its improvement of esthetic outcomes, experimental studies suggest that DAP may exhibit lower antimicrobial activity than TAP^74^. However, the available clinical evidence remains limited, precluding definitive conclusions regarding its comparative effectiveness in REPs.

Calcium hydroxide represents an alternative strategy that operates on a different biological premise. Compared with antibiotic pastes, it generally exhibits lower cytotoxicity and better preservation of stem cell viability while maintaining a suitable environment for cell proliferation^75^,^76^. However, its antimicrobial activity may be less effective against mature bacterial biofilms, and calcium hydroxide's high pH and its associated cytotoxic effects mean that direct contact with apical tissues should be avoided.^77^ It should therefore be placed in the canal carefully to avoid apical extrusion.

Recent systematic reviews have found no single intracanal medicament that clearly outperforms the others across all clinical outcomes. While antibiotic pastes are associated with increased dentinal wall thickening, calcium hydroxide has demonstrated higher rates of apical closure.^78^ Both TAP and calcium hydroxide have shown favorable clinical and radiographic outcomes, including symptom resolution and periapical healing.^79^,^80^ One review reported that TAP and calcium hydroxide produce comparable successful clinical outcomes, whereas the available evidence for DAP is relatively limited, precluding equally robust conclusions regarding its effectiveness.^74^ Overall, the current data suggest that both TAP and calcium hydroxide are suitable intracanal medicaments for REPs.^21^,^22^ The choice of which to use should be guided by the desired balance among antimicrobial efficacy, stem cell preservation, discoloration risk, and the specific clinical characteristics of each case.

Several experimental biomaterials, including multifunctional peptide hydrogels, antimicrobial hydrogels, chitosan, graphene oxide-copper nanocomposites, bioactive glass micro- and nanospheres, and silver nanoparticles, have also been investigated as potential alternatives to conventional intracanal medicaments.^81^ Experimental studies suggest that these materials can combine antimicrobial activity and bio film control with improved stem cell compatibility while promoting angiogenesis, odontogenesis and osteogenesis.^81^ However, these findings remain restricted to laboratory investigations, and clinical evidence supporting their incorporation into routine regenerative endodontic protocols is lacking.

Overall, the existing evidence suggests that persistent microorganisms and biofilms remain major obstacles to successful regeneration.^35^ The principal challenge, therefore, lies not simply in maximizing antimicrobial activity but in achieving sufficient disinfection while preserving the biological conditions necessary for stem cell survival and tissue regeneration.^34^ Future advances will likely depend on developing intracanal medicaments capable of simultaneously controlling infection and actively supporting regenerative processes.^82^

### Current clinically available scaffolds

The intentional induction of apical bleeding remains the cornerstone of contemporary REPs, providing an inexpensive, readily available autologous scaffold that is free of immunological concerns.^21^,^22^ The resulting blood clot forms a fibrin-rich matrix containing platelets and growth factors capable of recruiting mesenchymal stem cells (most likely originating from the apical papilla and periapical tissues), thereby supporting cell adhesion, migration, proliferation, and angiogenesis.^5^ Clinicians must recognize, however, that histological studies consistently demonstrate that blood clot-based REPs primarily result in tissue repair rather than the regeneration of a native pulp-dentin complex.^16^,^36^ Furthermore, significant clinical limitations persist, including difficulties in inducing sufficient apical bleeding,^11^,^42^ achieving adequate blood clot volume and position,^34^ and maintaining clot stability within the canal.^34^

When inducing a blood clot is not feasible, alternative autologous scaffolds (e.g., platelet-rich plasma [PRP], platelet-rich fibrin [PRF], platelet pellet [PP], and concentrated growth factor [CGF]) may be used.^83^ Obtained via centrifugation of the patient's blood, these materials provide a concentrated source of platelets and growth factors with minimal biological risk.

Among the currently available scaffolds, blood clot, PRP, and PRF remain the most widely used in clinical practice,^83^ producing comparable clinical and radiographic performance in terms of root length and thickness gain.^12^,^42^,^83^ Meta-analyses indicate that PRP and PRF yield clinical and radiographic outcomes for root maturation and periapical healing that are similar to those obtained with the blood clot, although the certainty of the available evidence remains constrained by the methodological quality of the studies included.^12^,^83^ While some systematic reviews suggest that PRP and PRF may improve short-term clinical outcomes, these advantages are not consistently maintained during long-term follow-up.^12^ CGF, which contains higher concentrations of growth factors such as epidermal growth factor (EGF), platelet-derived growth factor (PDGF), fibroblast growth factor (FGF), vascular endothelial growth factor (VEGF), and bone morphogenetic protein (BMP), has produced promising preliminary results regarding apical healing and root development^84^,^85^. Ultimately, however, current evidence does not justify the routine replacement of the blood clot with platelet concentrates, as their clinical superiority has yet to be definitively established.^12^,^83^

### Emerging biomaterials and experimental tissue engineering approaches

While autologous scaffolds remain the sole clinically established approach in REPs,^21^,^22^ numerous biomaterials have been developed to address their biological and mechanical limitations.^86^ Naturally derived polymers have attracted significant attention for their excellent biocompatibility, intrinsic bioactivity, and ability to mimic extracellular matrix components.^87^,^88^ These polymers include injectable collagen-based hydrogels, gelatin and gelatin methacryloyl (GelMA), hyaluronic acid, fibrin, alginate, chitosan, and chemically modified derivatives.^88^

Experimental studies indicate that these biomaterials provide favorable environments for stem cell adhesion, proliferation, differentiation, and angiogenesis.^88^ However, their rapid degradation, limited mechanical stability, and poorly controllable physicochemical properties pose significant challenges that often necessitate material modification through crosslinking or combination with other biomaterials.^81^

**Figure 2:**
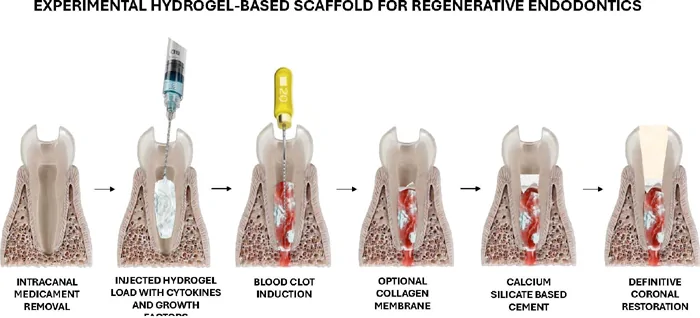
Experimental hydrogel-based scaffold for regenerative endodontics.

To date, clinical evaluations of these materials remain scarce. A randomized controlled clinical trial compared PRF, fibrin-chitosan hydrogel, fibrin hydrogel containing metronidazole and ciprofloxacin, and fibrin hydrogel containing chitosan alone within a cell-homing strategy.^89^ While no statistically significant differences in periapical healing were observed among the evaluated groups, the antibiotic-loaded fibrin-chitosan hydrogel demonstrated the highest overall healing rate.^89^ Consequently, despite encouraging preclinical data, the available clinical evidence remains insufficient to justify the routine incorporation of these biomaterials into REPs (Figure 2).

Schematic representation of an injectable hydrogel loaded with cytokines and growth factors designed to support endogenous stem cell recruitment. Although promising, this strategy remains predominantly experimental and has yet to be translated into routine clinical practice.

Synthetic biomaterials have also attracted research attention for their tunable physicochemical properties, reproducibility, and potential for large-scale manufacturing.^88^ Among these biomaterials, degradable poly(α-hydroxy esters), including poly(glycolic acid) (PGA), poly(lactic acid) (PLA), poly(lactic-co-glycolic acid) (PLGA), and poly(ε-caprolactone) (PCL), have demonstrated the capacity to support odontogenic stem cell viability and differentiation under experimental conditions.^88^,^90^,^91^ Despite these advantages, their lack of intrinsic bioactivity and the need to optimize their degradation kinetics remain critical barriers to clinical translation.^88^

Self-assembling peptides (SAPs) represent another promising class of scaffold materials because they spontaneously organize into nanostructured hydrogels that closely resemble the extracellular matrix.^92^ Their injectable nature facilitates delivery into the root canal system, and their biological properties promote cell adhesion,^93^ differentiation, and angiogenesis.^92^,^94^ However, their clinical applicability remains limited by challenges related to peptide sequence optimization, manufacturing complexity, and production costs, as well as regulatory approval.^88^

Three-dimensional bioprinting represents one of the most advanced tissue engineering strategies currently under investigation.^20^ Bioinks enable the incorporation of stem cells alongside growth factors,^20^,^95^ exosomes,^20^ immunomodulatory molecules,^20^ and other bioactive compounds^20^,^95^ into customized three-dimensional constructs^95^ designed to replicate the native pulp microenvironment.^21^ While these technologies represent an exciting future direction for regenerative endodontics, they remain in the nascent experimental stages and should be regarded as promising research strategies rather than clinically applicable therapies.^20^

Overall, current evidence supports blood clot, PRP, and PRF as the only scaffolding approaches backed by meaningful clinical experience.^83^ In contrast, while injectable biomaterials, synthetic scaffolds, and advanced tissue engineering technologies have demonstrated considerable biological promise, their validation remains primarily dependent on preclinical evidence.^88^ The successful translation of these technologies into routine clinical practice will require robust clinical trials that demonstrate not only their biological feasibility but also predictable patient-centered outcomes.

### Cell homing and cell-based transplantation

Two primary tissue engineering strategies have been proposed for regenerative endodontics: cell homing (cell-free) and cell-based transplantation.^96^,^97^ Importantly, all contemporary REPs currently performed in clinical practice are fundamentally based on the cell-homing concept.^13^ Rather than introducing exogenous cells into the root canal system, these procedures aim to recruit endogenous stem/ progenitor cells from the apical papilla and periapical tissues by combining growth factor release from dentin, induced bleeding, and scaffold formation.^5^,^46^ Consequently, cell homing represents the only tissue engineering strategy currently supported by meaningful clinical evidence in regenerative endodontics.^13^ Despite its clinical feasibility, this approach is biologically limited by its dependence on the patient's endogenous regenerative capacity, which may contribute to the variability observed in root maturation and tissue composition following REPs.^13^

**Figure 3:**
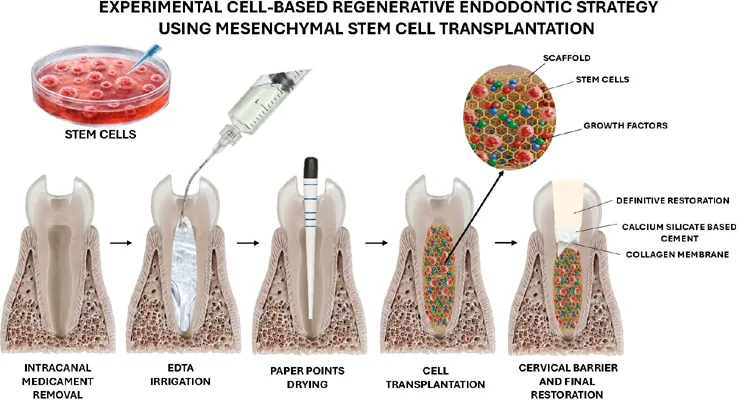
Experimental cell-based regenerative endodontic strategy using mesenchymal stem cell transplantation.

Conversely, cell-based transplantation aims to improve treatment predictability by introducing ex vivo expanded stem cells directly into the disinfected root canal, typically encapsulated within a prefabricated scaffold^98^ (Figure 3). Transplanted cells may be autologous or allogeneic and can be delivered either as heterogeneous cell populations or as selected stem cell subpopulations. Despite representing one of the most promising regenerative strategies, the clinical translation of cell transplantation remains limited.^96^,^97^ Beyond its operational complexity and high costs, widespread implementation faces substantial regulatory challenges.^96^,^97^ These include the need to comply with good manufacturing practice requirements and the need for specialized stem cell processing, banking facilities, and quality-control procedures, as well as uncertainties regarding reproducibility, long-term safety, and cost-effectiveness.^96^,^97^ Consequently, this approach remains restricted to experimental studies and early-phase clinical trials.^98^

Schematic representation of the isolation, ex vivo expansion, and transplantation of mesenchymal stem cells into a disinfected root canal within a biomaterial scaffold. Although promising, this strategy remains largely experimental and has yet to be incorporated into routine clinical practice.

Alternative tissue engineering strategies are also being investigated. Cellular aggregates, including spheroids, tissue strands, and cell sheets, enable cells to produce their own extracellular matrix, establishing a more physiological microenvironment that may improve cell differentiation, angiogenesis, and tissue organization.^99^ However, significant challenges remain regarding mechanical stability, manufacturing reproducibility, large-scale production, and cell viability following transplantation.^100^

Similarly, stem cell-derived extracellular vesicles and exosomes have emerged as promising cell-free therapeutic alternatives capable of modulating inflammation, promoting angiogenesis, and stimulating tissue regeneration through paracrine signaling.^101^ By avoiding many of the biological risks associated with cell transplantation, these strategies may offer important translational advantages. Nevertheless, standardizing isolation protocols, dosage, storage conditions, and quality control remain major obstacles to routine clinical application.^102^

While cell transplantation, engineered cellular constructs, and extracellular vesicle-based therapies represent compelling future directions for regenerative endodontics, contemporary clinical practice continues to rely almost exclusively on cell-homing strategies.^13^,^102^ Bridging the gap between these experimental technologies and routine patient care remains one of the major translational challenges in the field.^20^ Table 2 provides a comprehensive overview of current regenerative endodontic strategies, detailing their levels of evidence, clinical applicability, and the major challenges hindering their translation into routine clinical practice.

**Table 2:** Current regenerative endodontic strategies: level of evidence, clinical applicability, and translational challenges.

Strategy	Biological principle	Highest level of evidence	Clinical applicability	Translational maturity	Major limitations
Cell homing (blood clot)	Recruitment of endogenous stem/ progenitor cells after induced bleeding	Clinical guidelines, systematic reviews, meta-analyses	Routine clinical practice	Established	Variable root maturation; repair rather than true pulp-dentin regeneration
Platelet concentrates (PRP, PRF, CGF)	Delivery of autologous growth factors to enhance regeneration	Randomized clinical trials, systematic reviews	Clinically available	Established / Early adoption	No consistent superiority over blood clot; preparation variability
Calcium hydroxide, TAP and DAP *(intracanal medicaments)*	Canal disinfection while preserving regenerative potential	Clinical studies, systematic reviews	Routine clinical practice	Established	Biological trade-off between antimicrobial activity, stem-cell viability, discoloration and bacterial resistance
Novel irrigants (Ca(OCl)_2_, chitosan)	Improved antimicrobial activity with enhanced stem-cell compatibility	In vitro and animal studies	Experimental	Experimental	Lack of clinical validation
Adjunctive irrigation activation (PUI, XP-Endo Finisher, EDDY, SWEEPS)	Improve irrigant penetration and biofilm disruption	Laboratory studies with limited clinical evidence	Selectively used	Emerging	Limited evidence demonstrating superior clinical outcomes in REP
Natural hydrogels (collagen, fibrin, GelMA, hyaluronic acid, alginate, chitosan)	Biomimetic scaffold supporting cell migration and differentiation	Mainly preclinical studies; limited clinical trials	Experimental	Experimental	Mechanical instability, degradation, limited clinical validation
Synthetic scaffolds (PLA, PGA, PLGA, PCL, SAPs)	Controlled scaffold architecture and degradation	Laboratory and animal studies	Experimental	Experimental	Limited intrinsic bioactivity; manufacturing optimization required
Cell transplantation	Direct implantation of ex vivo expanded stem cells	Preclinical studies and early-phase clinical trials	Experimental	Experimental	GMP requirements, regulatory approval, cost, cell banking, reproducibility
Cell aggregates / spheroids / organoids	Three-dimensional cell organization with endogenous extracellular matrix production	Laboratory studies	Experimental	Experimental	Standardization, transplantation, cell survival and scalability
Extracellular vesicles / exosomes	Cell-free biological signaling and immunomodulation	Mainly preclinical studies	Experimental	Experimental	Isolation, dosage, storage stability and regulatory challenges
3D bioprinting	Patient-specific engineered pulp constructs	Laboratory studies	Experimental	Experimental	Bioink optimization, manufacturing complexity, regulatory approval and absence of clinical validation

### Sealing | restoration

Sealing and coronal restoration are fundamental components of REPs, as they preserve the biologically favorable environment established following canal disinfection and scaffold placement.^103^ The loss of the coronal seal can lead to bacterial recontamination, the persistence of residual microorganisms, and the disruption of the regenerative microenvironment, thereby compromising treatment outcomes.^103^ Consequently, contemporary REPs position statements recommend placing hydraulic calcium silicate cements (HCSCs) over the blood clot or scaffold to provide both an effective coronal seal and a biologically favorable interface with the underlying tissues.^21^,^22^ White MTA, calcium silicate-based bioceramics, and tricalcium silicate cements are the materials most commonly recommended, particularly in esthetically demanding cases.^21^

Among currently available HCSCs, MTA remains the reference material due to its excellent biocompatibility, antimicrobial activity, biological sealing ability, and capacity to modulate cytokine production while promoting cell migration and differentiation.^104^ However, tooth discoloration remains a clinically relevant concern, although it is less pronounced with white MTA than with gray MTA.^105^ It is also limited by its relatively long setting time, which increases the risk of material displacement or washout in the presence of blood and may delay placement of the definitive restoration.^104^ Calcium silicate-based materials with shorter setting times have therefore emerged as attractive alternatives, offering important practical advantages in contemporary clinical protocols.^104^

Other HCSCs have also demonstrated favorable biological properties, including calcium ion release,^104^ high biocompatibility, and the ability to promote the viability and odontogenic differentiation of stem cells from the apical papilla.^106^ Biodentine® (Septodont, Saint-Maur-des-Fossés, France), for example, has demonstrated low cytotoxicity, reduced potential for discoloration, a shorter setting time, and greater resistance to displacement during condensation.^104^,^107^ Although several calcium silicate-based materials exhibit promising biological and handling characteristics, robust comparative clinical evidence demonstrating the superiority of one material over another in regenerative endodontic procedures remains scarce.^104^

During REPs, the blood clot or scaffold is typically stabilized approximately 3-4 mm below the cementoenamel junction to provide adequate space for placement of the sealing biomaterial.^21^ A collagen membrane can be placed over the scaffold before the coronal barrier is placed. Although considered optional according to current AAE recommendations,^21^ limited evidence suggests that collagen membranes may improve localized dentinal wall thickening without significantly influencing the overall clinical or radiographic outcomes of REPs.^15^

Between treatment appointments, temporary coronal sealing should provide an effective barrier against bacterial leakage while maintaining adequate mechanical resistance.^108^ Glass ionomer cement remains a preferred temporary restorative material due to its adhesion to dentin, fluoride release, and biocompatibility.^109^ In cases requiring prolonged temporization, covering the glass ionomer with composite or flowable resin can further enhance the mechanical integrity and sealing ability of the temporary restoration.^21^

In conclusion, while the choice of sealing material may influence handling characteristics, esthetics, and biological properties, maintaining an effective and durable coronal seal is considerably more important for long-term REPs success^35^ than the selection of any specific calcium silicate-based material.

### Outcomes and success criteria

According to the AAE, the primary objective of REPs is to resolve clinical signs and symptoms as well as provide radiographic evidence of periapical healing.^21^ The secondary objective is continued root maturation, including increases in root length and dentinal wall thickness. Recovery of a positive response to pulp sensibility tests is considered a desirable outcome but is not required for treatment success, as it may reflect revascularization rather than true pulp regeneration.^17^ The ESE also considers the absence of inflammatory external root resorption, the preservation of the periodontal ligament, and the absence of discoloration as relevant treatment outcomes.^22^

These outcome measures are widely accepted; however, considerable heterogeneity exists in the methods used to assess REPs success. Root maturation, dentinal wall thickening, apical closure, and periapical healing have been evaluated using various imaging modalities and measurement protocols, limiting cross-study comparisons.^110^,^111^ Likewise, pulp sensibility responses should be interpreted with caution because available tests assess neural responses rather than the histological nature or functional integrity of the newly formed tissue.^14^,^83^,^112^ Patient-reported outcomes, including pain, oral health-related quality of life, esthetic satisfaction, and treatment experience, are rarely assessed in REPs studies despite constituting important measures of treatment success from the patient's perspective.^110^ Outcome definitions, imaging protocols, and assessment methodologies should therefore be standardized to improve the quality and comparability of future clinical studies.^111^,^113^,^114^

Clinical studies consistently report high survival rates, resolution of apical periodontitis, and excellent tooth retention following REPs, with outcomes comparable to those achieved by contemporary apexification procedures.^14^,^115^ However, continued root maturation remains considerably more variable, with reported rates ranging from 1.6% to 76.2% for root lengthening,^5^,^42^,^116^,^117^ 4% to 79.2% for dentinal wall thickening,^5^,^42^,^116^,^117^ and 32% to 91% for apical closure.^33^,^42^,^112^

These favorable clinical outcomes should not be interpreted as evidence of complete biological regeneration. Histological studies consistently demonstrate that the tissues formed following contemporary REPs rarely replicate the architecture of the native pulp-dentin complex.^65^ A systematic review of animal and human studies reported that newly formed intracanal tissues predominantly consist of fibrous connective tissue, cementum-like tissue, and bone-like tissue rather than organized pulp tissue.^118^ Similarly, histological analyses of immature human teeth have shown that newly formed tissue most often signifies repair, or a combination of repair and regeneration, rather than complete regeneration of the original pulp-dentin complex.^119^ Consequently, clinical success, radiographic healing, root maturation, and even the recovery of pulp sensibility should not be utilized as surrogate markers of true histological regeneration or the restoration of a functional pulp-dentin complex.^16^,^119^ This distinction is crucial for interpreting the current evidence and highlights a major biological limitation of contemporary regenerative endodontics.

Persistent infection remains the most frequently reported cause of treatment failure, accounting for 79% of cases.^35^ External inflammatory root resorption (particularly in traumatized teeth).^11^,^35^ tooth discoloration,^104^,^117^ and revascularization-associated intracanal calcification^116^,^117^ are additional complications that can compromise treatment outcomes.^35^ Although intracanal calcification generally requires no intervention in asymptomatic teeth, conventional root canal treatment with guided access may become necessary should symptoms or apical disease develop.

The management of failed REPs is challenging due to variations in clinical presentation dictated by the etiology, stage of root development, timing of failure, and intracanal changes.^35^ Retreatment options include a second REPs, apexification, conventional root canal treatment, endodontic microsurgery, or extraction, but current recommendations are based primarily on case reports and case series, and robust evidence-based retreatment guidelines remain absent.^120^,^121^ Consequently, retreatment decisions should be tailored to the cause offailure, the stage of root development, the restorability of the tooth, and patient-related factors until more robust clinical evidence becomes available.

Finally, a major limitation to improving REPs outcomes is the difficulty of translating in vitro data to human applications due to challenges with animal experimental models. While rodents such as rats and mice are commonly used in etiopathological research,^122^,^123^ their small size limits their application for testing treatment approaches and protocols. Larger animals such as dogs,^36^,^124^ pigs,^125^ and ferrets^126^ are therefore considered for these purposes, but due to ethical considerations and high costs, their involvement in REPs research is restricted.^127^ Consequently, it is important to recognize that the gap between in vitro studies and clinical trials requires the continual improvement of experimental designs and planning for future research.^127^

## Conclusion

Regenerative endodontic procedures demonstrate favorable clinical outcomes and represent a valuable treatment option for immature necrotic teeth. However, current protocols primarily promote tissue repair rather than the true regeneration of a functional pulp-dentin complex, and important biological and clinical challenges remain unaddressed. While numerous emerging tissue engineering strategies show promise in laboratory and preclinical studies, their translation into routine clinical practice remains hindered by scarcity of high-quality clinical evidence, lack of standardized treatment protocols and outcome measures, and significant regulatory, manufacturing, and implementation challenges. Consequently, bridging the gap between experimental innovation and predictable, evidence-based clinical application remains a major priority for the future of regenerative endodontics.

## Data Availability

The datasets generated during and/or analyzed during the current study are available from the corresponding author on reasonable request.
